# Surgical Approach in Management of Acute Appendicitis Within a De Garengeot Hernia: A Case Report

**DOI:** 10.7759/cureus.80669

**Published:** 2025-03-16

**Authors:** Suanne C MacConnell, Joel D Stein

**Affiliations:** 1 General Surgery, St John of God Hospital Midland, Perth, AUS

**Keywords:** appendicitis, de garengeot hernia, femoral hernia, mcevedy approach, primary closure

## Abstract

De Garengeot hernias are rare surgical occurrences and even more so when the histopathology reveals acute appendicitis. Varying approaches are documented with regards to open versus laparoscopic and the use of mesh. This case demonstrates the successful use of an open high McEvedy approach with primary suture closure in an elderly female who presented with an acute history of a right groin lump, right iliac fossa pain, nausea and fever.

## Introduction

De Garengeot hernia is an eponymous name with an incidence ranging from 0.5-5% [1-6], describing the presence of the appendix within a femoral hernia. The incidence is even less (0.1%) if the appendix demonstrates acute inflammation [1-2,5,7]. The pre-operative diagnosis can be unreliable from clinical symptoms alone, but the use of cross-sectional imaging can be utilised to prepare for an appropriate surgical approach [1,2] and assist in assessing for complications including perforation and abscess formation [8]. Varying techniques including open vs laparoscopic and primary closure vs mesh repair are reported in the literature without a clear consensus [3,5,7,9]. Although many case reports are published there remains lacking evidence towards a standardised approach [4-7,10].

## Case presentation

History of presenting complaint

An 84-year-old female presented with a three-day history of non-radiating right iliac fossa (RIF) pain associated with nausea, reduced oral intake, subjective fevers and loose stools. The patient denied constitutional symptoms and or any weight loss. The patient had a significant background of previous Mirizzi’s syndrome resulting in an open procedure and formation of a Roux-en-Y in 2017 as well as polymyalgia rheumatica, interstitial lung disease, type 2 diabetes mellitus and recurrent urinary tract infections. She was also admitted previously in 2021 with a partial small bowel obstruction which was managed conservatively. On review by the surgical team in the emergency department the patient was found to be afebrile and haemodynamically stable with a heart rate of 85 bpm and a blood pressure of 140/95 mmHg. She was tender with guarding in the RIF with further findings of rebound tenderness and Rovsing’s positive. There was a tender palpable mass in her right groin.

Investigations

**Figure 1:**
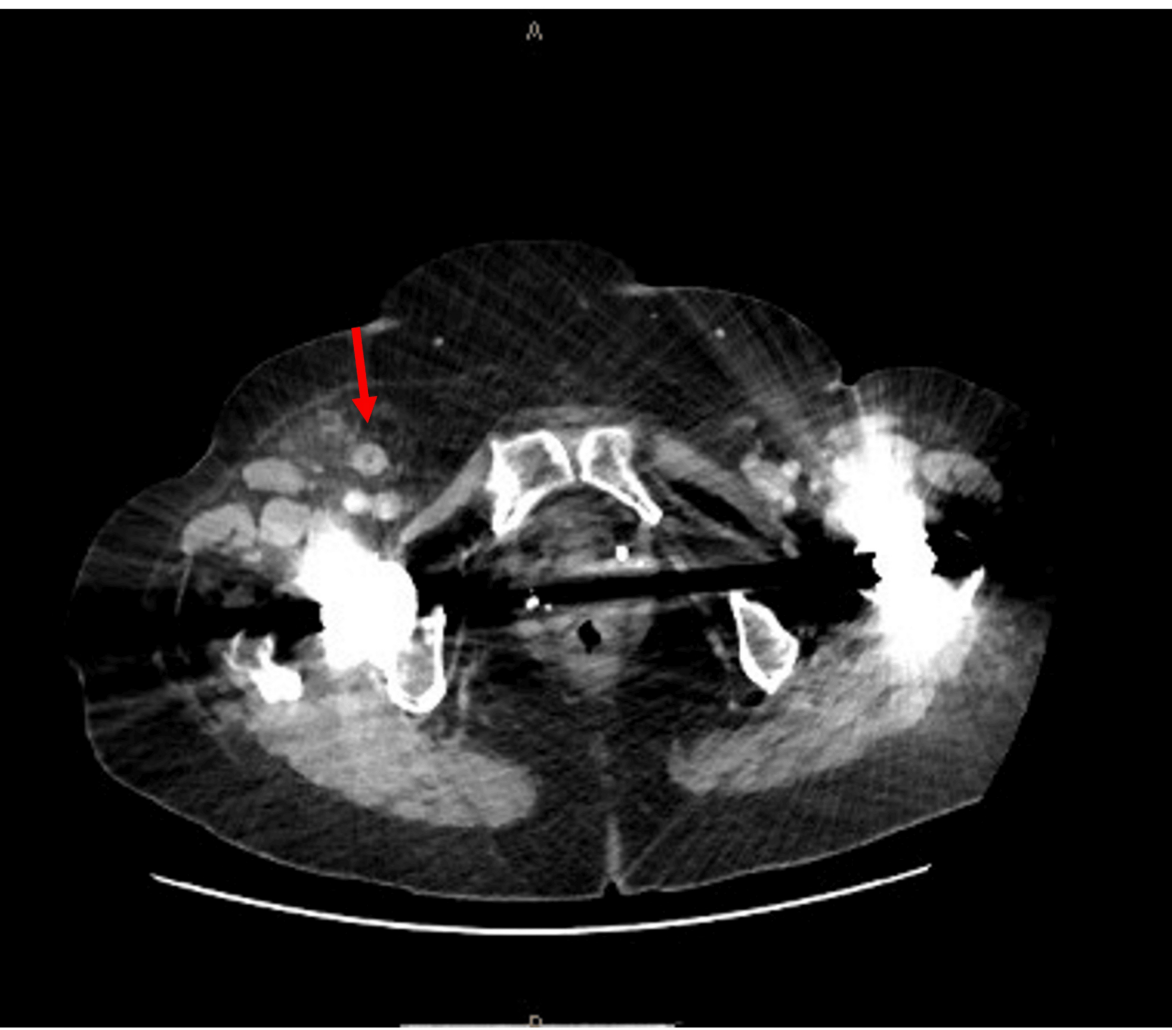
Axial view of acute appendicitis within right femoral hernia (red arrow)

**Figure 2:**
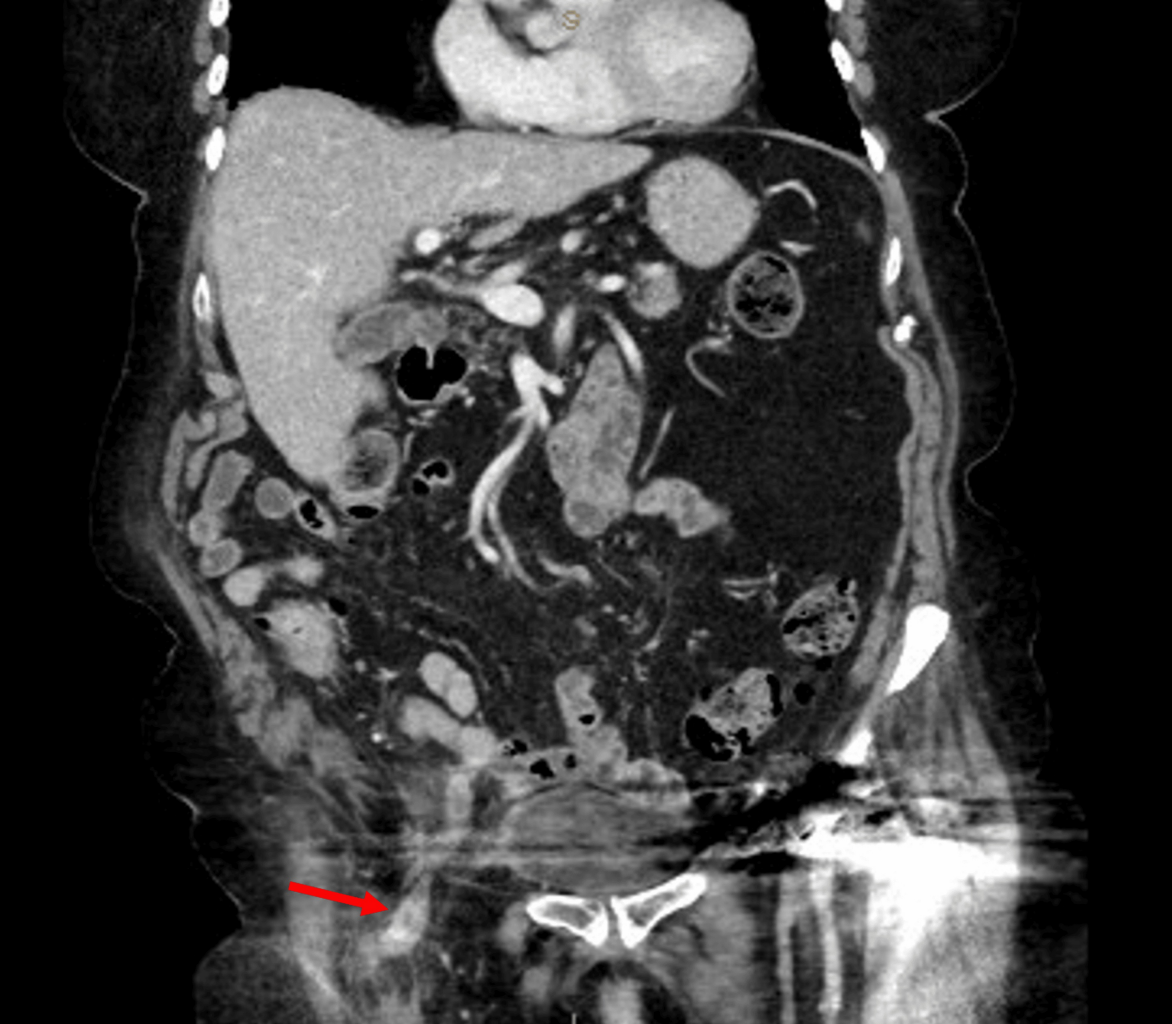
Coronal view demonstrating thickened inflamed appendix within right femoral hernia (red arrow)

Her biochemical markers revealed a white cell count of 12.1 x 10^9^/L and a C-reactive protein of 78.7 mg/L (Table 1). A computed tomography (CT) abdomen/pelvis was performed given the patient’s age and clinical findings which revealed a right femoral hernia containing mesenteric fat as well as a thick-walled inflamed appendix (Figures 1, 2). No evidence of focal perforation or drainable collection was identified. A decision was made to proceed to theatre where an open right primary suture femoral hernia repair and appendicectomy was performed.

**Table 1:** Biochemical markers (elevated)

	Patient	Normal range
White cell count (WCC) [x10^9^/L]	12.1	4.5-11
C-reactive protein (CRP) [mg/L]	78.7	<3

Treatment and outcome

A high McEvedy open approach was undertaken secondary to the patient’s age, co-morbidities and body habitus. It was felt by the surgical team that this technique would allow for greater access to the intraperitoneal cavity if required. The sac was reduced and the appendiceal artery was controlled with suture ligation using a 2-0 vicryl. A purse-string technique with 3-0 PDS was utilised to secure the appendiceal base. Primary suture closure with 0-Ethibond was performed to approximate the femoral canal. The patient was reviewed by the surgical team on day 1 post-operatively and reported to be feeling better. She was examined and felt suitable for discharge with oral analgesia and antibiotics, with an outpatient clinic follow-up arranged.

Follow-up

At her six-week follow-up, the patient reported no concerns with pain or wound closure. On examination, the wound had healed and there was no palpable mass or evidence of acute failure/recurrence. Histopathology revealed acute appendicitis with no sinister features.

## Discussion

A De Garengeot hernia has a female predominance (81.1%) and a mean age of 69.8 years [2-7, 10-11]. As with this case report, the most common symptoms consist of groin tenderness and a groin bulge with a palpable groin mass observed in 95% of occasions [4, 6-7, 10-13]. De Garengeot hernias can pose an element of a surgical dilemma however the use of pre-operative abdominal CT imaging proves useful in reducing the cognitive load regarding the surgical approach [1-4,6,11]. The literature however reports the effective use of CT in establishing a diagnosis in less than a third of cases [10,13]. The debate continues regarding an open vs laparoscopic approach, however, it is understood that both an appendicectomy and hernia repair should be performed at the index emergency operation [1-3,5,7,12]. The most common surgical approach is through a groin incision [1,3,9-10] with a high McEvedy, Lotheissen transinguinal and Lockwood-low being described as the approaches of choice [2], noting that the appendix base must be accessible for the open approach to be successful [3,8]. Primary repair with a non-absorbable suture, as was performed in this case report, was deemed the most common hernia repair [5,7,9,11,13]. Laparoscopic approaches have been described in the literature and may prove useful if the diagnosis remains unclear because of an atypical presentation or inconclusive imaging at the time of operation, given it allows exploration of the abdominal cavity [2-3,8]. This approach, however, may prove challenging in providing adequate control of infection at the groin site as evident by the presence of groin erythaema [4,8,10], given this area is not explored or debrided via the laparoscopic approach. Intra-abdominal sepsis and subsequent peritonitis are uncommon secondary to the anatomical features of the femoral canal [2,5,7] suggesting that laparoscopic washout is unlikely. The use of mesh for the repair is reported to halve the risk of recurrence [3-4], however, placement of mesh poses a higher morbidity in the presence of contamination [4,11,13] but successful repair utilising mesh in the presence of inflammation is reported [1]. It is therefore recommended to assess the balance of infection status against that of a recurrence and place mesh accordingly [4]. In this case, the infection risk was deemed higher than recurrence, hence a primary closure was performed. Complications following the repair are recognised in 9.5% of cases with most pertaining to surgical-site infections [2,5-7,10] and rarely necrotising fasciitis secondary to perforation [2,6-8]. Regardless of the approach or closure technique, early treatment is recommended to improve outcomes and avoid complications [5-6,11]. Ultimately the approach and use of mesh comes down to surgeon preference, utilising a case-by-case basis, acknowledging that the finding may be unexpected and therefore prove more complex in managing [1,3,13].

## Conclusions

De Garengeot hernias pose a rare opportunity for surgical repair given the low incidence. Laparoscopic versus open approaches are likely dependant on multiple factors including patient background and surgeon preference. We present a case of a successful open approach with primary closure in a De Garengeot hernia with acute appendicitis. Surgeons should be aware of different anatomical approaches and closure techniques and decide management based on a case-by-case basis.
